# Neoadjuvant Therapy for Pancreatic Ductal Adenocarcinoma: Where Do We Go?

**DOI:** 10.3389/fonc.2022.828223

**Published:** 2022-06-16

**Authors:** Chenqi Wang, Guang Tan, Jie Zhang, Bin Fan, Yunlong Chen, Dan Chen, Lili Yang, Xiang Chen, Qingzhu Duan, Feiliyan Maimaiti, Jian Du, Zhikun Lin, Jiangning Gu, Haifeng Luo

**Affiliations:** ^1^ Department of Hepatobiliary Surgery, The First Affiliated Hospital of Dalian Medical University, Dalian, China; ^2^ Department of Oncology, The First Affiliated Hospital of Dalian Medical University, Dalian, China; ^3^ Department of General Surgery, The First Hospital of Northwest University (Xi’an No. 1 Hospital), Xi’an, China; ^4^ Department of Pathology, The First Affiliated Hospital of Dalian Medical University, Dalian, China

**Keywords:** neoadjuvant chemotherapy, neoadjuvant chemoradiotherapy, borderline resectable, locally advanced, FOLFIRINOX, gemcitabin

## Abstract

The incidence of pancreatic ductal adenocarcinoma (PDAC) has been on the rise in recent years; however, its clinical diagnosis and treatment remain challenging. Although surgical resection remains the only chance for long-term patient survival, the likelihood of initial resectability is no higher than 20%. Neoadjuvant therapy (NAT) in PDAC aims to transform the proportion of inoperable PDACs into operable cases and reduce the likelihood of recurrence to improve overall survival. Ongoing phase 3 clinical trial aims to validate the role of NAT in PDAC therapy, including prolongation of survival, increased R0 resection, and a higher proportion of negative lymph nodes. Controversies surrounding the role of NAT in PDAC treatment include applicability to different stages of PDAC, chemotherapy regimens, radiation, duration of treatment, and assessment of effect. This review aims to summarize the current progress and controversies of NAT in PDAC.

## Background

According to the American Cancer Society, pancreatic cancer will become the third leading cause of cancer-related deaths in the United States, whereas in China, the mortality rate is also increasing and ranked sixth among malignant tumors (1, 2). It remains one of the most malignant upper gastrointestinal tract tumors, and the 5-year overall survival (OS) rate fluctuates at 11%, owing to late diagnosis and low response to limited treatment options (2). Although surgical resection is central in the treatment of pancreatic ductal adenocarcinoma (PDAC), adjuvant chemotherapy has been shown to improve OS, progression-free survival (PFS), and disease-free survival (DFS) postoperatively. However, no more than 20% of patients with PDAC have the chance to undergo surgery because of late diagnosis; thus, studies have focused on improving the therapeutic effect in the remaining 80%.

PDAC is currently considered to be a systematic disease, and tumor local aggression, including some potential early micrometastasis, is the main reason for relapse after resection and even hard to be detected preoperatively. The application of neoadjuvant therapy (NAT) could reduce tumor burden and improve probability of R0 resection, which currently plays an important role in PDAC treatment. In addition, NAT has been proven to be effective in various malignancies, such as colorectal, breast, and esophageal cancers (3–5). NAT attempts to resect locally advanced PDAC through tumor shrinkage and elucidation of the tumor local aggression potential early micrometastasis. Simultaneously, NAT was also reported to test the biological behavior and eliminate the “undetectable” micrometastasis in borderline and resectable PDAC before surgery to prevent recurrence and prolong OS and DFS.

Although some scholars have indicated that NAT is beneficial for treating PDAC at all stages, some controversies remain. The results of OS, PFS, and DFS after surgery following NAT vary between investigations, possibly attributed to different chemotherapy regimens. Furthermore, the response rate, chemotherapy duration, reassessment standard, and operation timing are also pertinent factors that are being assessed in clinical trials. This review aimed to present current studies on NAT in different stages of PDAC, including resectable, borderline resectable, locally advanced, and other debates in this field.

## Neoadjuvant Therapy in Borderline Resectable and Locally Advanced Pancreatic Cancer

Borderline resectable pancreatic cancer (BRPC) and locally advanced pancreatic cancer (LAPC) are both absolute indications for NAT, according to the National Comprehensive Cancer Network (NCCN). Essentially, the definitions of BPRC and LAPC are somewhat unclear, because this definition is based on subjective morphology and imageological examination, such as “the encasement of celiac trunk or superior mesenteric artery no more than 180°”. Compared to RPC, it is difficult to achieve R0 resection in LAPC and BRPC; thus, application of NAT in these two types aims to increase resectability and further prolong OS. Owing to the late cancer stage, the purpose of NAT is to eliminate tumor cells and potential micrometastasis in the circulating blood, reduce the risk of postoperative recurrence, shrink the local tumor, and transform some inoperable tumors into operable ones. Nevertheless, there is still some debate regarding these circumstances.

BRPC is technically easier to resect; however, the NCCN guidelines have recommended NAT instead of upfront surgery for its treatment since 2016. In addition, the main controversy focuses on the following aspects (1): Does neoadjuvant chemotherapy reduce the recurrence rate? (2) Does neoadjuvant chemotherapy increase the R0 resection rate and the proportion of LN negative patients and prolong survival? (3) Which neoadjuvant chemotherapy regimen is best recommended? Therefore, OS, progression-free time, disease-free time, recurrence time, R0 resection rate, and negative lymph node rate were the main evaluation indicators.

LAPC, which accounts for 40% proportion of patients with PDAC at diagnosis, is technically unresectable, and therefore, it is incorrect to use the term NAT for this stage. However, 15%–69% of patients with LAPC have been reported to undergo resection after upfront chemotherapy (6–10). Therefore, in this manuscript, we will refer to chemotherapy for LAPC as NAT as well. The focus of NAT in LAPC is as follows: (1) The selection of chemotherapy regimens. Because LAPC occurs at a late stage with obvious peri-invasion or vascular encasement, intensive chemotherapy with at least two regimens is administered, and some can be combined with radiotherapy. However, which combination is the most effective? (2) The conversion rate and OS, which indicates whether NAT, following surgical resection, is more effective than the entire process of chemotherapy, because failure or no response of NAT for LAPC is equivalent to adjuvant chemotherapy in metastatic PDAC to some extent.

Multiple clinical trials suggest that NAT for BRPC and LAPC is effective, tolerable, and clearly improves OS (11, 12). In 2018, the first prospective, multi-center, randomized controlled phase II/III clinical trial including 58 patients with BRPC from South Korea found that the NAT group, which used gemcitabine-based chemoradiotherapy, had a longer median survival and higher R0 resection rate than the upfront surgery group (median survival: 21 months vs. 12 months, p = 0.028; R0 resection rate: 51.8% vs. 26.1%, p = 0.004). Furthermore, the trial was terminated early, owing to its huge advantage (13). In addition, a small, single-center, single-arm retrospective study from Germany showed that 14 patients with LAPC used oxaliplatin, irinotecan, fluorouracil, and leucovorin (FOLFIRINOX) as a neoadjuvant chemotherapy regimen; the conversion rate of surgery was 29%, the R0 resection rate was 75%, and the median OS was 31 months (14). Other large multicenter, prospective randomized clinical trials, such as the Prep-02/JSAP-05 (Japan, 2019) and PREOPANC (Netherlands, 2020), also confirmed the survival benefit of NAT in patients with BRPC (15, 16). However, some scholars are concerned about whether chemotherapy delays surgical resection and instead causes tumor progression in possible surgical candidates. Moreover, many studies support that recurrence of pancreatic cancer is due to its biological behavior itself, as it has poor response to chemotherapy (17). The time window offered by NAT is beneficial to weed out rapidly progressive cases, which often have poor tumor biology and have minimal benefit from surgical resection. Therefore, NAT requires a certain degree of screening.

**Figure 1:**
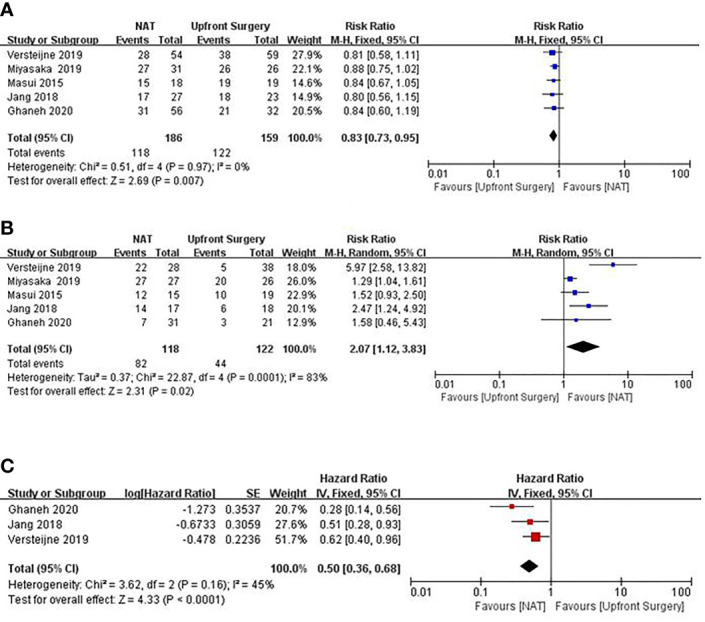
Forest plots showing risk ratios of resection rate **(A)**, R0 resection rate **(B)**, and HR of overall survival **(C)** in BRPC.

Some meta-analyses have demonstrated the advantages of NAT for BRPC and LAPC. Cloyd’s meta-analysis (18) results showed that, based on an intention-to-treat analysis, NAT resulted in improved OS compared to upfront surgery (HR, 0.73; 95% CI, 0.61–0.86). Although the overall resection rate was similar [risk ratio (RR) = 0.93; 95% CI, 0.82–1.04; I^2^ = 39.0%], NAT increased the likelihood of R0 resection (RR = 1.51, 95% CI 1.18–1.93, I^2^ = 0%) and negative lymph nodes (RR = 2.07; 95% CI, 1.47–2.91; I^2^ = 12.3%). Results of Xu’s meta-analysis (19) showed that FOLFIRINOX as a first-line treatment has a significant downstaging effect on patients with LAPC or BRPC, with an R0 resection rate of 40%, a median OS of 15.5 to 35.4 months, and a median PFS of 10.0 to 27.1 months. These favorable results can be attributed not only to improved surgical skills and perioperative managementbut also to NAT-controlled potential micrometastasis and surgical resection. We used Forest plots to demonstrate the effect of the NAT and upfront surgery for BRPC. The resection rate in upfront surgery group was higher than in NAT group (RR 0.83, 95%CI: 0.73–0.95; I^2^ = 0%; P = 0.007) in **Figure 1A**. However, the R0 resection rate in NAT group was significantly higher than in upfront surgery group (RR 2.07; 95%CI: 1.12–3.83; I^2^ = 83%; P = 0.02) in **Figure 1B**. The pooled HR for OS of NAT compared to upfront surgery was 0.50 (95% CI 0.36–0.68, I^2^ = 45%; p < 0.01) in **Figure 1C**, the pooled HR remained significantly in favor of NAT. Although the resection rate was higher in the upfront surgery group, the NAT group had a higher R0 resection rate and longer OS. Hence, NAT is therefore often recommended in an attempt to eradicate occult systemic disease, facilitate margin-negative (R0) resection, maximize OS, and spare patients with evolving metastasis otherwise futile surgery.

In terms of chemotherapy regimen, the concern is different for BRPC and LAPC. For BRPC, although technically resectable, upfront surgery may make it difficult to achieve R0 resection and early recurrence is possible. The main role of NAT is to achieve R0 resection, control potential micrometastasis, and prolong the OS, which is the focus of the regimen. FOLFIRINOX is usually recommended as first-line therapy with so-called “high efficacy and high toxicity.” In 2018, a single-arm phase II clinical trial enrolled 48 patients with BRPC. Thirty-four patients completed FOLFIRINOX plus radiotherapy, 32 underwent surgery, and 31 achieved R0 resection. The median PFS of patients undergoing surgery was 48.6 months, the 2-year PFS rate was 55%, and the 2-year survival rate was 72%. However, the regimen often leads to grade 3/4 neutropenia, thrombocytopenia, neuropathy, and other adverse reactions (20). Although FOLFIRINOX has high efficacy, its toxicity remains relatively high, especially among Asian populations (21, 22).

Therefore, the modified FOLFIRINOX (mFOLFIRINOX) regimen was developed with a similar effect and relatively less toxicity, and its effect has been validated to be better than gemcitabine monotherapy as adjuvant chemotherapy for PDAC. The A021101 trial confirmed the safety and efficacy of the mFOLFIRINOX regimen combined with radiotherapy for BRPC. A total of 22 patients were included in the study, 15 of whom underwent surgery. Of the 15 surgical patients, 14 underwent R0 resections. The median survival time of the 22 patients was 21.7 months (23). The other gemcitabine-based therapies plus nab-paclitaxel or S1 are currently widely applied with satisfactory effects. Williams et al. (24) compared the results of mFOLFIRINOX with nab-paclitaxel/Gemcitabine for chemotherapy regimen and found that mFOLFIRINOX had a higher response rate, longer DFS time, and fewer positive lymph nodes, but the median OS time was not significantly different (33.3 vs. 27.1 months, p = 0.105). A large, multicenter, retrospective study by Macedo et al. (25) included 274 patients with pancreatic cancer who underwent radical surgery after NAT and compared the therapeutic effects of FOLFIRINOX and gemcitabine. The results showed that the overall R0 resection rate after NAT was 82.5%, and the median survival time was 32 months. The results of the subgroup analysis showed that there was no statistically significant difference between the two chemotherapy regimens on the prognosis of patients, and they were both feasible options. The chemotherapy cycle that patients can tolerate is a prognostic risk factor; patients who insist on completing the seven-cycle FOLFIRINOX regimen had a significant survival advantage compared to those who only received the short-course chemotherapy (48.0 months vs. 31.2 months, p < 0.05). One study from a Japanese group reported that NAT with gemcitabine plus S1 could significantly improve survival time and R0 resection rate with comparable complications, whereas the operation time and blood loss were even shorter and less in the NAT group than in the upfront surgery group (26). Another study even indicated that an early switch to nab-paclitaxel/gemcitabine from FOLFIRINOX failure patients may help increase the treatment response (27), which prompts the fact that these two regimens may involve different mechanisms. Nevertheless, without considering the patient’s physical condition, in addition to improved R0 rates, effective systemic NAT is also important for improving survival. In this sense, it is possible that NAT with FOLFIRINOX, which has the expense of higher toxicities, could improve overall oncologic outcomes.

For LAPC, we are more concerned with the tumor conversion rate, because conversion failure is equivalent to chemotherapy for late-stage pancreatic cancer, whereas some “barely” resectable PDACs eventually became unresectable. Therefore, we need to consider which chemotherapy regimen has the highest conversion rate, because it implies higher probability of R0 or/and R1 resection which may get longer survival time. In existing reports, the FOLFIRINOX regimen seems to be the commonly used first-line regimen. Multiple studies have shown the modest effect of the FOLFIRINOX regimen. Murphy et al. (10) conducted a single-arm phase II study that used FOLFIRINOX plus losartan and radiotherapy as neoadjuvant chemotherapy regimens for LAPC. The results showed that it can downstage LAPC and attain a conversion rate of 69%, as well as a R0 resection rate of 61%. Among the patients who underwent resection, the median PFS was 21.3 months, and the median OS was 33.0 months (10). For example, Hackert et al. (6) reported 125 patients with locally advanced PDAC who were treated with FOLFIRINOX in a NAT setting. The resection rate was 61%, the median OS after resection was 16.0 months, and FOLFIRINOX was confirmed to be independently associated with a favorable prognosis (6) Besides FOLFIRINOX, some investigators add other treatment or drugs, such as radiotherapy, capecitabine and losartan, which seems to have higher R0 resection rate and longer OS. The benefit of addition of radiation or losartan to chemotherapy needs to be further explored in larger clinical trials. In response to the strong effects and high toxicity of FOLFIRINOX, 41 patients with LAPC from the study by Liang et al. in China (9) received mFOLFIRINOX, and the objective response rate of the tumor was 37.1% and the surgical conversion rate reached 34.1%. The median OS of the patients who underwent transformation surgery was 27.7 months, and the median PFS was 19.3 months; correspondingly, the median OS and PFS of patients with LAPC who did not receive neoadjuvant chemotherapy were only 8.9 and 7.6 months, respectively (9). Currently, there are no clear guidelines on which neoadjuvant systemic protocol is better to increment resection rates and survival in patients with LAPC. However, fewer gemcitabine-based chemotherapy regimens have been reported compared to FOLFIRINOX. According to the results of FOLFIRINOX compared to gemcitabine as adjuvant chemotherapy for patients with PDAC after resection and considering the advanced stage of the tumor in patients with LAPC, stronger systematic chemotherapeutics are more recommended and may have higher efficacy; probably, this maybe one of the reasons why the results from the group by Murphy et al. were more satisfied than the group by Hackert et al. In this sense, it is possible that neoadjuvant treatment with FOLFIRINOX may have higher efficacy than others; however, more RCTs are urgently needed to be clarify this hypothesis (28–30). However, in the largest phase II study (LAPACT) reported in 2018, 107 patients with LAPC who received a nab-paclitaxel/gemcitabine regimen had a resection rate of only 15% and an R0 resection rate of 44% (7). The NEOLAP trial compared nab-paclitaxel/gemcitabine and nab-paclitaxel/gemcitabine sequential FOLFIRINOX as the LAPC neoadjuvant chemotherapy regimen (8). The results indicated that the two regimens did not show statistical differences in the primary and secondary endpoints, and both can achieve the chance of R0/R1 surgical resection for at least 30%. In addition, the nab-paclitaxel/gemcitabine sequential FOLFIRINOX regimen showed greater advantages in the conversion rate and median OS (8). Hence, larger randomized trials are needed to explore the advantages and disadvantages of these two regimens.

Chemotherapy remains the core of NAT. Radiotherapy, as a local treatment method, can effectively relieve pain symptoms inpatients and strengthen local tumor control. However, whether radiotherapy improves patient outcomes remains unclear. Therefore, it remains debatable whether neoadjuvant chemotherapy should be combined with radiotherapy.

Many studies have shown that neoadjuvant chemoradiotherapy is tolerable and safe and can effectively reduce tumor remnants on the side of the involved blood vessel and significantly increases R0 resection (31–33). A retrospective study indicated that the neoadjuvant chemoradiotherapy group had a higher negative rate of lymph nodes (53% vs. 23%, p < 0.01) and R0 resection rate (91% vs. 79%, p < 0.01) but a similar median OS (33.6 vs. 26.4 months, p = 0.09) compared to the neoadjuvant chemotherapy group (34). Furthermore, neoadjuvant chemoradiotherapy is effective in increasing the conversion and resection rates of LAPCs (35–37). In contrast, the PREOPANC-1 study used gemcitabine-based chemoradiotherapy as the neoadjuvant regimen for BRPC and showed that the experimental group had a higher R0 resection rate (71% vs. 40%, p < 0.001) and longer median DFS than the upfront surgery group (8.1 months vs. 7.7 months p = 0.032) (16). Similarly, Tran et al. (38) concluded that neoadjuvant chemoradiotherapy can benefit patients with BRPC. However, the studies from different centers may be different because there is no universal definition of BRPC. For example, the criteria included in the study by Tran et al. (38) use the NCCN guidelines for selection of patients with BRPC, whereas the study by Murphy et al. (20) depended on “multidisciplinary committee” mode, which was more dependent on experience. This also implies that the standard of BPRC needs to be normalized in the future.

Nonetheless, the main problem is that these studies have only shown that neoadjuvant chemoradiotherapy strategies are superior to upfront surgery strategies. Currently, no studies have been able to answer the question of the administration sequence of these two parts. Currently, European medical centers are mostly based on neoadjuvant chemotherapy, whereas the United States is more inclined toward combined chemoradiotherapy. Stereotactic radiation therapy and intensity-modulated radiation therapy are increasingly used as NATs in patients with BRPC and LAPC. However, few studies have evaluated the effect of these modalities on surgical resection. Further clinical trials are needed to evaluate the efficacy of radiotherapy for BRPC and LAPC.

In conclusion, NAT could improve the R0 resection rate as well as prolong OS in patients with BRPC and LAPC. Although there is no consensus on the selection of neoadjuvant regimens for BRPC and LAPC, FOLFIRINOX seems to be the first-line recommended regimen for BRPC and LAPC; however, nab-paclitaxel or S1 plus gemcitabine are more often used in Asian countries with satisfactory effects and higher tolerance. Combined radiotherapy may improve the conversion rate, but this requires further investigation. The published and ongoing clinical trials are summarized in **Tables 1**, **2**.

**Table 1:** Neoadjuvant therapy in LAPC.

Type of Study	DOI	Reference	Treated With Radiotherapy	Total	Stage of Disease	Treatment Regimen	Resection Rate	R0 Resection Rate	Median OS (months)	Median DFS (months)
Clinical trial	10.1001/jamaoncol.2019.0892.	Murphy et al. (10)	YES	49	LAPC	FOLFIRINOX + losartan + radiotherapy	80.9%	88.2%	31.4	NR
Clinical trial	10.1038/bjc.2016.45	Stein et al. (39)	YES	31	LAPC	mFOLFIRINOX + radiotherapy	41.9%	100%	26.6	17.8
Retrospective study	10.1245/s10434-014-4225-1	Blazer et al. (40)	YES	25	LAPC	FOLFIRINOX + radiotherapy	44.0%	90.9%	21.2	NR
Retrospective study	10.1002/jso.23392	Boone et al. (41)	YES	13	LAPC	FOLFIRINOX + radiotherapy	20.0%	50.0%	NR	NR
Observational study	10.1245/s10434-014-3898-9	Marthey et al. (42)	YES	77	LAPC	FOLFIRINOX + radiotherapy	36.4%	89.3%	22.0	NR
Retrospective study	10.1097/coc.0000000000000349	Wo et al. (43)	YES	74	LAPC	FOLFIRINOX and gemcitabine + radiotherapy	39.2%	NR	18.1	14.9
Retrospective study	10.1097/SLA.0000000000001850	Hackert et al. (6)	YES	575	LAPC	FOLFIRINOX + radiotherapy	60.8%	NR	16.0	NR
						gemcitabine + radiotherapy	46.6%		16.5	
						other regimens	51.6%		14.0	
Retrospective study	10.1245/s10434-015-4647-4	Sadot et al. (44)	YES	101	LAPC	FOLFIRINOX and gemcitabine + radiotherapy	30.7%	51.6%	25.0	NR

LAPC, locally advanced pancreatic cancer; FOLFIRINOX, oxaliplatin, irinotecan, fluorouracil, and leucovorin; mFOLFIRINOX, modified FOLFIRINOX; OS, overall survival; DFS, disease-free survival; NR, not reported.

**Table 2:** Neoadjuvant therapy in BRPC.

Type of study	DOI	Reference	Treated With Radiotherapy	Total	Stage of Disease	Treatment Regimen	Resection Rate	R0 Resection Rate	Median OS (months)	Median DFS (months)
Clinical trial	10.1200/JCO.2020.38.15_suppl.4505	Ghaneh et al. (45)	NO	32	BRPC	upfront surgery	65.6%	14.3%	NR	NR
			NO	20		gemcitabine capecitabine	55.4%	22.6%	NR	NR
			NO	20		FOLFIRINOX				
			YES	16		chemoradiotherapy				
Clinical trial	10.1200/JCO.19.02274	Versteijne et al. (16)	YES	54	BRPC	gemcitabine + radiotherapy	51.9%	78.60%	17.6	6.3
			NO	59		upfront surgery	64.4%	13.20%	13.2	6.2
Clinical trial	10.1016/j.ijrobp.2019.08.057.	Tran et al. (38)	YES	25	BRPC	FOLFIRINOX + radiotherapy + gemcitabine	52.0%	100.0%	24.4	NR
Clinical trial	10.1007/s00280-016-3121-8	Okada et al. (46)	NO	10	BRPC	mFOLFIRINOX	70.0%	71.40%	NR	NR
Clinical trial	10.1097/SLA.0000000000002705	Jang et al. (13)	YES	27	BRPC	gemcitabine + radiotherapy	63.0%	82.4%	21.0	NR
			NO	23	BRPC	upfront surgery	78.3%	33.3%	12.0	NR
Clinical trial	10.1001/jamasurg.2016.1137.	Katz et al. (23)	YES	22	BRPC	mFOLFIRINOX + capecitabine + radiotherapy	68.2%	93.3%	21.7	NR
Clinical trial	10.1001/jamaoncol.2018.0329	Murphy et al. (20)	YES	48	BRPC	FOLFIRINOX + radiotherapy	66.7%	96.9%	37.3	NR
Clinical trial	10.1007/s00595-016-1310-z	Masui et al. (26)	NO	18	BRPC	gemcitabine + S1	83.30%	80.0%	21.7	NR
				19	BRPC	upfront surgery	100.0%	52.6%	21.1	NR
Retrospective study	10.1245/s10434-014-3486-z	Rose et al. (47)	NR	53	BRPC	gemcitabine + docetaxel	58.5%	87.1%	23.6	NR
Retrospective study	10.1007/s11605-018-3966-8	Javed et al. (48)	YES	151	BRPC	Fluorouracil-based + radiotherapy	63.6%	77.1%	23.7	NR
						Fluorouracil and gemcitabine-based + radiotherapy				
						gemcitabine-based + radiotherapy				
						others				
Retrospective study	10.1002/jso.21954	Patel et al. (49)	YES	14	BRPC	gemcitabine + docetaxel + capecitabine + 5-FU + radiotherapy	64.3%	88.9%	15.64	10.48
Retrospective study	10.4174/astr.2017.93.4.186	Kim et al. (50)	YES	40	BRPC	gemcitabine based + radiotherapy	85.0%	76.5%	20.0	NR
						5-FU + radiotherapy				
						FOLFIRINOX + radiotherapy				
Retrospective study	10.1016/j.surg.2019.05.010	Barnes et al. (51)	YES	185	BRPC	FOLFIRINOX + radiotherapy	62.2%	96.5%	20.0	19.0
						gemcitabine/nab-paclitaxel + radiotherapy				
						Others				
Retrospective study	10.1245/s10434-019-07309-8	Miyasaka et al. (52)	NO	57	BRPC	gemcitabine + nab-paclitaxel	87.1%	100%	43.9	NR
						upfront surgery	100%	76.9%	23.1	NR
Retrospective study	10.1016/j.suronc.2017.08.003	Ielpo et al. (53)	YES	26	BRPC	gemcitabine and nab-paclitaxel + radiotherapy	61.5%	NR	18.9	NR
			NO	19		upfront surgery	100%	NR	13.5	NR

BRPC, borderline resectable pancreatic cancer; FOLFIRINOX, oxaliplatin, irinotecan, fluorouracil, and leucovorin; mFOLFIRINOX, modified FOLFIRINOX; OS, overall survival; DFS, disease-free survival; NR, not reported.

## Neoadjuvant Therapy in Resectable Pancreatic Cancer

Resectable pancreatic cancer (RPC) is an irrefragable indication for surgery. The current treatment for RPC remains controversial as to whether direct surgery or NAT should be chosen. However, the reason why NAT is still recommended in RPC is based on the following points: (1) suppression of primary tumors and elimination of potential micrometastasis that are not visible upon preoperative imaging; (2) reducing tumor volume and increasing R0 resection rate; (3) screening biological behaviors and providing individualized treatment; and (4) ability to start chemotherapy sooner than potential surgery especially for cancer wherein early metastasis is frequent and known. Pancreatic cancer itself is a chemotherapy-insensitive tumor, and the period of chemotherapy may lead to loss of opportunity for surgery, especially in non-responsive cases. Furthermore, the choice of chemotherapy regimen, response assessment, chemotherapy cycle, and timing of surgery remain inconclusive.

Nonetheless, results from randomized controlled trials conducted globally are worth considering. The first multicenter, open-label, randomized controlled phase II–III clinical trial (PACT-15) from Italy with 88 patients with RPC showed that the administration of the PEXG regimen (cisplatin, epirubicin, gemcitabine, and capecitabine) extended the patient’s median OS time to 38.2 months, which validated the efficacy of NAT in RPC (54). The phase II/III Prep-02/JSAP-05 trial (Japan, 2019) also showed that, compared to upfront surgery, NAT with gemcitabine plus S1 could offer a significant benefit with a median OS of 36.7 vs. 26.6 months, respectively (15). The PREP-01 study (Japan, 2018), which also applied gemcitabine plus S1, had similar results to the above-mentioned trial (55). Another trial is currently evaluating the role of NAT in RPC (Alliance 021806; https://clinicaltrials.gov/ct2/show/NCT04340141). The results are worth considering.

With regard to toxicity, FOLFIRINOX has been the recommendation in LAPC, BRPC, and even metastatic PDAC. Recently, the SWOG S1505 trial conducted by Ahmad et al. suggested that the tolerability and safety of these two schemes were good with acceptable toxicity; however, no significant difference was found among these two groups in terms of the median OS and DFS time. The study did not demonstrate an improved OS with NAT, compared with historical data from adjuvant trials in RPC. However, it demonstrated the feasibility of multidisciplinary treatment using NAT for patients with RPC (56). Another randomized phase II trial showed that the combination of gemcitabine and cisplatin had a more significant effect than gemcitabine monotherapy (57). In addition, Bradley’s meta-analysis (58) supported that neoadjuvant chemotherapy can increase the R0 resection rate and the 1-, 3-, and 5-year survival rates. The above results indicated that NAT could extend OS in patients with RPC, whereas combined therapy is more effective than monotherapy.

Because of the insensitivity of pancreatic cancer to chemotherapy, neoadjuvant chemotherapy may lead to tumor progression and loss of the opportunity for surgery. Hence, the question is, can patients benefit from this treatment? As mentioned earlier, the current definition of BRPC is mainly based on morphology and imageological examination without considering systemic conditions. Therefore, the NCCN guidelines do not directly recommend neoadjuvant chemotherapy for all patients with RPC. Instead, the guidelines recommend it for patients with high-risk factors, such as significant weight loss, severe abdominal pain that indicates segmental ganglion invasion, high levels of CA19-9, and enlarged lymph nodes that are highly suspicious for metastasis (16, 59). Can RPCs with these five categories be summed up in the BRPC to some extent? Except for five cases, for other RPCs, despite the benefits of NAT, is it better than postoperative adjuvant chemotherapy? In the APACT phase III trial, patients who received nab-paclitaxel/gemcitabine regimen as adjuvant chemotherapy after resection reached a relatively higher median OS of 40.5 months, and GEM regimen reached a mOS of 36.2 months (60). Therefore, it is important to consider whether such survival can be achieved with NAT.

**Figure 2:**
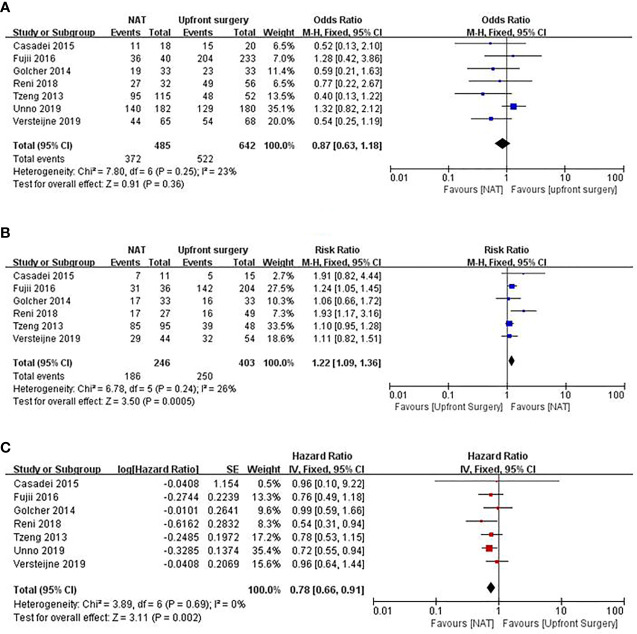
Forest plots showing risk ratios of resection rate **(A)**, R0 resection rate **(B)**, and HR of overall survival **(C)** in RPC.

A multicenter prospective phase II clinical trial enrolled 59 patients with RPC who were administered gemcitabine plus bevacizumab and radiotherapy; however, 7.9% had tumor progression, which means that they may lose the opportunity for longer survival (12). One clinical trial conducted by Casadei et al. (61) was terminated early because a substantial number of patients withdrew from the trial due to the delay in the timing of the operation, whereas no significant difference in R0 resection rate between the surgery alone and neoadjuvant chemoradiotherapy groups was found in the completed cases. Although the results of PREOPANC suggest that neoadjuvant chemoradiotherapy can increase the R0 resection rate of patients with RPC compared with upfront surgery, there was no significant difference in the OS between the two groups (16). Golcher et al. (62) and Casadei et al. (61) also believed that neoadjuvant chemotherapy neither improved the prognosis significantly nor increased the R0 resection rate and the negative rate of lymph nodes. The meta-analysis by Zhan et al. (63) included 14 studies on NAT for RPC, and the results showed that it failed to benefit the patients. Other meta-analysis also supported that neoadjuvant chemotherapy could improve the R0 resection rate but did not prolong OS (64, 65). We used Forest plots to demonstrate the effect of the NAT and upfront surgery for RPC. There was no significant difference in resection rate among the two groups (RR 0.87, 95% CI 0.63–1.18, I^2^ = 23%; p = 0.36) in **Figure 2A**. On the other hand, among patients who underwent resection, NAT increased the likelihood of an R0 resection (RR 1.22, 95% CI 1.09–1.36, I^2^ = 26%; p = 0.0005) in **Figure 2B**. The pooled HR for OS of NAT compared to upfront surgery was 0.78 (95% CI 0.66–0.91, I^2^ = 0%; p = 0.002) in **Figure 2C**; the pooled HR remained significantly in favor of NAT. NAT was associated with a higher R0 resection rate and a longer survival time than upfront surgery.

In general, although NAT may increase the R0 resection rate and reduce postoperative recurrence and positive rate of local lymph nodes. However, whether it can improve OS and DFS needs to be further investigated. Published and ongoing clinical trials are presented in **Table 3**.

**Table 3:** Neoadjuvant therapy in RPC.

Type of study	DOI	Reference	Treated With Radiotherapy	Total	Stage of Disease	Treatment Regimen	Resection Rate	R0 resection Rate	Median OS (months)	Other Outcome Measure		Median DFS (months)
Clinical trial	10.1097/SLA.0000000000004155	Ahmad et al. (56)	NO	55	RPC	mFOLFIRINOX	87%	85%	22.4	2-year OS rate	41.60%	10.9
			NO	47		gemcitabine/nab-paclitaxel	77%	85%	23.6		48.80%	14.2
Clinical trial	10.1200/JCO.2019.37.4_suppl.189	Michiaki et al. (15)	NO	182	RPC	gemcitabine + S1	NR	NR	36.7	NR		NR
			NO	180		upfront surgery	NR	NR	26.6			
Clinical trial	10.1200/JCO.19.02274	Versteijne et al. (16)	YES	65	RPC	gemcitabine + radiotherapy	68%	66%	14.6	NR		9.2
			NO	68		upfront surgery	79%	59%	15.6			9.3
Clinical trial	10.1097/SLA.0000000000004535	Takahashi et al. (66)	YES	51	RPC	radiotherapy + S1	NR	NR	37.7	2-year PFS rate	45%	NR
			NO	51		gemcitabine + S1	NR	NR	NR		55%
Clinical trial	10.1097/SLA.0000000000000251	O’Reilly et al. (67)	NO	38	RPC	gemcitabine + oxaliplatin	71%	74%	27.2	18-month survival rate	63%	NR
Clinical trial	10.1245/s10434-019-07735-8	Eguchi et al. (68)	YES	63	RPC	gemcitabine + S1 + radiotherapy	86%	100%	55.3	1-year survival rate	83.30%	NR
Clinical trial	10.1007/s00066-014-0737-7	Golcher et al. (62)	YES	33	RPC	gemcitabine + cisplatin + radiotherapy	58%	89%	17.4	NR		NR
			NO	33		upfront surgery	70%	70%	14.4		
Observational study	10.1007/s00535-016-1217-x	Fujii et al. (69)	YES	40	RPC	5-FU + oteracil + gimeracil + S-1 + radiotherapy	92%	97%	24.9	NR		NR
			NO	233		upfront surgery	88%	70%	23.5		
Clinical trial	10.1016/S2468-1253 (18)30081-5	Reni et al. (54)	NO	32	RPC	cisplatin + epirubicin + gemcitabine + capecitabine	90%	63%	38.2	3-year survival rate	55%	NR
			NO	56		upfront surgery	87.5%	56.5%	NR		NR
Randomized Controlled Trial	10.1007/s11605-015-2890-4	Casadei et al. (61)	YES	18	RPC	gemcitabine + radiotherapy	61.1%	38.9%	22.4	NR		NR
			NO	20		upfront surgery	75.0%	25.0%	19.5
Retrospective study	10.1007/s11605-013-2412-1	Tzeng et al. (70)	YES	115	RPC	gemcitabine and cisplatin + radiotherapy	83.0%	89.5%	28.0	NR		NR
			NO	52		upfront surgery	92.3%	81.3%	25.3
Retrospective study	10.1016/j.suronc.2017.08.003	Ielpo et al. (53)	YES	19	RPC	gemcitabine and nab-paclitaxel + radiotherapy	78.9%	NR	22.1	NR		21
			NO	17		upfront surgery	100%	NR	24.8			14

RPC, resectable pancreatic cancer; FOLFIRINOX, oxaliplatin, irinotecan, fluorouracil, and leucovorin; mFOLFIRINOX, modified FOLFIRINOX; OS, overall survival; PFS, progression-free survival; NR, not reported.

## Neoadjuvant Therapy and Comprehensive Therapy

The application of immunotherapy in pancreatic cancer has been studied in recent years. Findings show that immune monotherapy, such as anti-PD-L1 or PD-1, has only a slight response in PDAC. This may be attributed to the dense matrix of the pancreas and the highly immunosuppressive tumor microenvironment. On the basis of this information, some researchers have found that combined radio- and immunotherapy in patients with metastatic PDAC could improve treatment sensitivity (71). Although immunotherapy has progressed in basic research and has been attempted in clinical practice, its effect is still unsatisfactory, and current research results show that immunotherapy alone cannot improve the prognosis of pancreatic cancer (72). At present, larger numbers of clinical trials related to immunotherapy combined with radiotherapy are conducted in PDAC, such as NCT03563248, NCT02305186, and NCT03161379. However, the long-term effects need to be studied further.

In addition to immunotherapy, targeted therapy is another important option for pancreatic cancer. The Kirsten rat sarcoma viral oncogene homolog (KRAS) mutation reaches more than 95% in patients with PDAC, and the origin of Sotorasib (AMY510) terminates the undruggable period of the oncogene. However, this therapy mainly targets KRAS G12C instead of the hotspot of G12D in PDAC. The initial results of the application of adagrasib (KRYSTAL-1 trial), another KRAS G12C inhibitor, were reported in the ASCO GI Cancer Symposium in 2021, with a 100% disease control rate; however, this is only a single-arm trial with a small sample size (n = 10) that needs further analysis. Other related targets are under evaluation in clinical trials (73, 74). One representative trial is the POLO study, a phase III clinical trial that confirmed that advanced pancreatic cancer patients carrying germline BRCA1/2 gene mutations can benefit from maintenance therapy with the poly adenosine diphosphate-ribose polymerase inhibitor, olaparib, after platinum-based chemotherapy (75). Although the results of the POLO trial have supported the clinical benefit of olaparib maintenance therapy in a subgroup of patients with germline BRCA-mutated and metastatic pancreatic cancer, targeted therapy for pancreatic cancer has not yet achieved a breakthrough.

Similar to the G12C mutation, the proportion of patients with pancreatic cancer having germline BRCA mutations is limited. Moreover, the most common KRAS mutation in pancreatic cancer has no effective targeted drugs for clinical use. Therefore, we also speculated whether olaparib could be used as a NAT. Studies have also shown that epidermal growth factor receptor and mitogen-activated extracellular signal-regulated kinase inhibitors have radiotherapy sensitization effects (76–78). Therefore, targeted therapy may enhance the effects of radiotherapy and play a role during NAT. In addition, Cuneo et al. (79) found that in patients with LAPC, AZD1775 combined with neoadjuvant chemoradiotherapy could improve the OS compared with neoadjuvant chemoradiotherapy alone in comparison to historical control. This study demonstrates the importance of targeted NAT.

Although immunotherapy and targeted therapy combined with neoadjuvant chemotherapy are theoretically valuable, their actual effects need to be further investigated.

## Neoadjuvant Therapy and Reassessment

The timing of surgery after NAT is a point for discussion, because the final aim of NAT is surgical resection. During NAT, patients need to be reevaluated for resectability to check the progress of the disease and formulate the next treatment plan. At present, there is still a lack of ideal methods for reassessment in clinical practice, and the most commonly used is the Response Evaluation Criteria in Solid Tumors (RECIST). In addition, classifying borderline resectable tumors is based on morphology; therefore, noninvasive imaging is preferred. However, neoadjuvant radiotherapy can induce local inflammation and fibrosis, making it difficult to determine the response. Studies have shown that the accuracy of computed tomography in predicting the R0 resection rate after NAT is only approximately 71% (80). Furthermore, some tumors can achieve resection even if they have no manifestations of downstaging on imaging; thus, a new reassessment indicator for resectability is needed. In addition, the panel recommends that adopt standardized imaging reporting template for preoperative staging of pancreatic cancer to improve surgical decision. For accurate disease staging, the panel also recommends that all patients who have no obvious metastatic disease or extensive local invasion at initial routine abdominal CT examinations undergo a repeat examination with dedicated pancreas protocol multidetector CT angiography (81). The maximum standard uptake value of the tumor on positron emission tomography–computed tomography and the change in the ultrasound echo intensity of the tumor after NAT may become new modes of assessment (80). In addition, some studies have shown that the change in CA-199 levels after NAT is also an important indicator of resectability (82, 83). Katz et al. (84) confirmed the value of CA199 for predicting resection after NAT in patients with resectable disease and found that patients with the borderline resectable disease who experienced a decrease of CA19-9 >50% during NAT had higher odds or R0 margin status (OR: 4.2, P = 0.05). Indeed, a decrease in CA199 during NAT was associated with improved OS (85). However, other scholars found that the normalization of CA19-9 after NAT has a stronger prognostic value than breadth of the reduction (86–88). However, no consensus has been reached on the threshold value of CA199, and more clinical trials are needed to prove it. Further, the detection of circulating tumor DNA, circulating tumor cells, and exosomes through liquid biopsy can monitor treatment response and disease progression in patients with PDAC (89). It is currently believed that patients receiving NAT should undergo surgical exploration if there is no evidence of disease progression upon imaging assessment (90). Although invasive methods are harmful to patients, it is worth trying when imaging is difficult to conduct. However, reassessment approaches for PDAC after NAT are still not standardized. Therefore, the development of multi-technologies such as imagingomics and pathomics is expected to provide a more comprehensive and scientific reassessment protocol of NAT for pancreatic cancer.

## Neoadjuvant Therapy and Surgical Opportunity

Although the timing of surgery after NAT remains inconclusive, most prospective studies recommend 4 to 8 weeks after NAT. This treatment strives for surgical opportunities for patients and influences their physical and immune state, which decreases their endurance against the operation. There are two aspects of the impact of NAT on surgical complications: the reduction in tumor volume reduces the difficulty of surgical resection, and NAT causes inflammation and fibrosis of the tissues surrounding the tumor, increasing the risk of intraoperative bleeding and adjacent tissue damage. In terms of postoperative complications, NAT can reduce the incidence of clinically related pancreatic fistula; however, there is no significant difference in the incidence of other complications, such as gastric emptying disorder (91). Although the situation may become more complicated when targeted immunotherapy is administered, this is based on studies that have small samples or those that are retrospective. Therefore, further research is needed to confirm these findings.

## Conclusion

The treatment of patients with localized PDAC is being transformed from surgery to integrated therapy. NAT in these patients appears to be crucial to improve likelihood of R0 resectability and survival compared to adjuvant therapy; however, confirmatory phase 3 trials are ongoing. FOLFIRINOX is often the regimen of choice for NAT; however, clinical trials directly comparing these regimens have not shown any difference. Several additional questions remain unanswered, including the role of radiation and novel therapeutics, duration of NAT, and reassessment standards.

## Author Contributions

JG, GT, and HL contributed to conception and design of the study. CW, JZ, YC, LY, XC, and QD organized the database. CW and JG wrote the first draft of the manuscript. JD, ZL, and FM wrote sections of the manuscript. DC, HL, and GT supervised the manuscript. All authors contributed to the article and approved the submitted version.

## Conflict of Interest

The authors declare that the research was conducted in the absence of any commercial or financial relationships that could be construed as a potential conflict of interest.

## Publisher’s Note

All claims expressed in this article are solely those of the authors and do not necessarily represent those of their affiliated organizations, or those of the publisher, the editors and the reviewers. Any product that may be evaluated in this article, or claim that may be made by its manufacturer, is not guaranteed or endorsed by the publisher.
